# Right atrial wall inflammation detected by ^18^F-FDG PET/CT may be significantly associated with persistent atrial fibrillation: a prospective case-control study

**DOI:** 10.1186/s12872-023-03592-2

**Published:** 2023-11-30

**Authors:** Peng Wan, Bing Wang, Wenji Yu, Li Shang Zhai, Bo Qian, Feifei Zhang, Bao Liu, Jianfeng Wang, Xiaoliang Shao, Yunmei Shi, Qi Jiang, Meng Fei Wang, Shan Shao, Yuetao Wang

**Affiliations:** 1 Department of Cardiology, The Third Affiliated Hospital of Soochow University, No.185, Juqian Street, Changzhou, Jiangsu Province, 213003 China; 2 Department of Nuclear Medicine, The Third Affiliated Hospital of Soochow University, No.185, Juqian Street, Changzhou, Jiangsu Province, 213003 China; 3 Institute of Clinical Translation of Nuclear Medicine and Molecular Imaging, Soochow University, Changzhou, Jiangsu Province, China

**Keywords:** Atrial fibrillation, PET-CT scan, Glucose metabolism, Heart, Inflammation

## Abstract

**Aim:**

Atrial fibrillation (AF) is a progressive disease from paroxysmal to persistent, and persistent AF (PerAF) had worse prognosis. AF has potential link with inflammation, but it is not clear whether PerAF or paroxysmal AF (ParAF) is more closely related to inflammation. On the basis of inhibiting myocardial physiological uptake, ^18^F-fluorodeoxyglucosepositron emission tomography/computed tomography (^18^F-FDG PET/CT) is an established imaging modality to detect cardiac inflammation. We aimed to decipher the association between AF and atrial inflammatory activity by ^18^F-FDG PET/CT.

**Methods:**

Thirty-five PerAF patients were compared to age and sex matched ParAF group with baseline ^18^F-FDG PET/CT scans prior to radiofrequency catheter ablation (RFCA) in the prospective case-control study. High-fat and low-carbohydrate diet and prolonged fast (HFLC+Fast) was applied to all AF patients before PET/CT. Then 22 AF patients with positive right atrial (RA) wall FDG uptake (HFLC+Fast) were randomly selected and underwent HFLC+Fast+heparin the next day. The CHA2DS2-VASc score was calculated to evaluate the risk of stroke. Clinical data, ECG, echocardiography, and atrial ^18^F-FDG uptake were compared.

**Results:**

PerAF patients had significantly higher probability of RA wall positive FDG uptake and higher SUVmax than ParAF group [91.4% VS. 28.6%, *P* < 0.001; SUVmax: 4.10(3.20–4.90) VS. 2.60(2.40–3.10), *P* < 0.001]. Multivariate logistic regression analyses demonstrated that RA wall SUV_max_ was the independent influencing factor of PerAF (OR = 1.80, 95%CI 1.02–3.18, *P* = 0.04). In 22 AF patients with RA wall positive FDG uptake (HFLC+Fast), the “HFLC+Fast+Heparin” method did not significantly change RA wall FDG uptake evaluated by either quantitative analysis or visual analysis. High CHA2DS2-VASc score group had higher RA wall ^18^F-FDG uptake [3.35 (2.70, 4.50) vs, 2.8 (2.4, 3.1) *P* = 0.01].

**Conclusions:**

RA wall FDG positive uptake was present mainly in PerAF. A higher RA wall ^18^F-FDG uptake was an independent influencing factor of PerAF. RA wall FDG uptake based on ^18^F-FDG PET/CT may indicate pathological inflammation.

**Trial registration:**

http://www.chictr.org.cn, ChiCTR2000038288.

## Introduction

Atrial fibrillation (AF), which is the most common arrhythmia in clinical practice, is a progressive disease that manifests as a transition from paroxysmal to persistent AF [1]. Compared with paroxysmal AF (ParAF), persistent AF (PerAF) is more prone to stroke, which is the most serious complication in patients with AF [2]. Clinical evidence suggests a link between inflammation and AF, but as a progressive disease, it is not clear whether PerAF or ParAF is more closely related to inflammation. Inflammation may affect the persistence of arrhythmias through participating in the electrical and structural remodeling associated with AF [3]. It was reported that by right atrial biopsy in AF patients, 66.7% patients had lymphomononuclear infiltration with adjacent cardiomyocyte necrosis, which suggested that right atrial inflammation is related to AF [4]. AF patients had increasing CRP and Interleukin-6 [5]. Multiple studies of community-based and clinical trial cohorts have clearly demonstrated that circulating biomarkers including hemodynamic stress (i.e., natriuretic peptides), inflammation (i.e., C-reactive protein), myocardial injury (i.e., cardiac troponin), and coagulation activity (i.e., D-dimer) improve risk prediction of AF incidence [4, 6, 7]. Pathological biopsy is the gold standard for the diagnosis of inflammatory activity. However, a pathological biopsy is invasive and cannot provide dynamic evaluation. Plasma inflammatory markers lack specificity and cannot fully reflect the local cardiac inflammation. Besides, discerning inflammation of the atrial wall with existing radiological methods presents a formidable task.

^18^F-fluorodeoxyglucosepositron emission tomography/computed tomography (^18^F-FDG PET/CT) is an established imaging modality to detect local inflammation [8]. The uptake degree of ^18^F-FDG, quantitatively measured by the maximum standardized uptake value (SUV_max_), is positively correlated with the density of inflammatory cells and reflects the inflammatory activity of tissues [9]. ^18^F-PET/CT has been successfully employed to visualize inflammatory activity in AF patients, with controversial results. Watanabe et al. showed that atrial FDG uptake was associated with AF and found macrophage and lymphocyte infiltration in FDG uptake by pathology, indicating that ^18^F-PET/CT may be a non-invasive tool for detecting atrial local inflammation [10]. Epicardial adipose tissue (EAT) was the source of inflammatory cytokines, and a retrospective study found that increased FDG activity of EAT, was independently associated with the enhanced FDG uptake in atrial wall [11]. However, some studies suggested that the uptake of 18F-FDG in cardiac PET/CT imaging is non-specific. Philipp S. Lange et al. suggested a generally increased myocardial glucose metabolism rather than a genuinely higher inflammatory activity in AF patients [12, 13].

Previous studies based on ^18^F-FDG PET/CT mainly focused on atrial FDG uptake in AF patients [10], while evidence of the relationship between the type of AF and RA wall FDG uptake is scarce. Besides, most of them were retrospective studies, inflammatory activity in the atria may be hampered by the physiological FDG uptake or myocardial energy substrate metabolism in heart when PET/CT performed under routine conditions. Heparin combined with prolonged fast could further exclude interference from energy substrate metabolism then confirm pathological inflammatory uptake.

This prospective case-control study aimed to determine the relationship between RA wall FDG uptake, ParAF and PerAF, reveal whether atrial uptake favor pathological inflammatory uptake rather than myocardial energy substrate metabolism, and the potential clinical significance of RA wall FDG uptake.

## Methods

### Study population

**Fig. 1:**
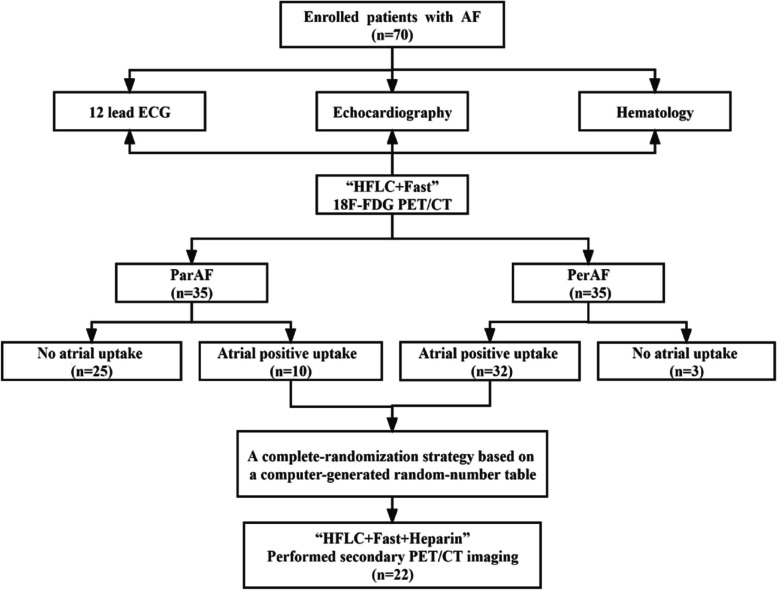
Study population

This prospective case-control study was conducted at the Third Affiliated Hospital of Soochow University, from January 2020 to September 2021, with approval by the institution’s ethics review committee [approval No: ChiCTR2000038288]. Patients admitted to cardiology department due to AF were continuously included. Diagnosis of AF was determined by cardiologist according to medical history or electrocardiogram (12-lead electrocardiogram or 24-h Holter electrocardiogram). According to the guidelines [14], ParAF is defined as a duration of less than 7 days, which can be converted to sinus rhythm spontaneously; PerAF is defined as a duration of more than 7 days, which often requires electrical or drug conversion. Course of diagnosis and disease duration: confirmed by diagnosis report after which disease duration was determined after comparing with study date. The exclusion criteria are as follows: 1. Individuals having previously undergone ablation for AF. 2. Patients who tested positive for COVID-19. 3. Patients diagnosed with thyroid disease. 4. Patients with a known or established diagnosis of sarcoidosis, pulmonary hypertension, or CAD, which could affect FDG uptake in the atrium were excluded from the study. This information was gathered through thorough review of patients’ medical histories and available health records prior to their inclusion in the study. 5. Patients diagnosed with any form of rheumatologic disease were also omitted from the study. Finally, 35 PerAF and 35ParAF with age- and gender- matched were enrolled. The flow chart was shown in Fig. 1. All participants provided written informed consent. The investigation conforms with the principles outlined in the Declaration of Helsinki.

### ^18^F-FDG PET/CT imaging

**Fig. 2:**
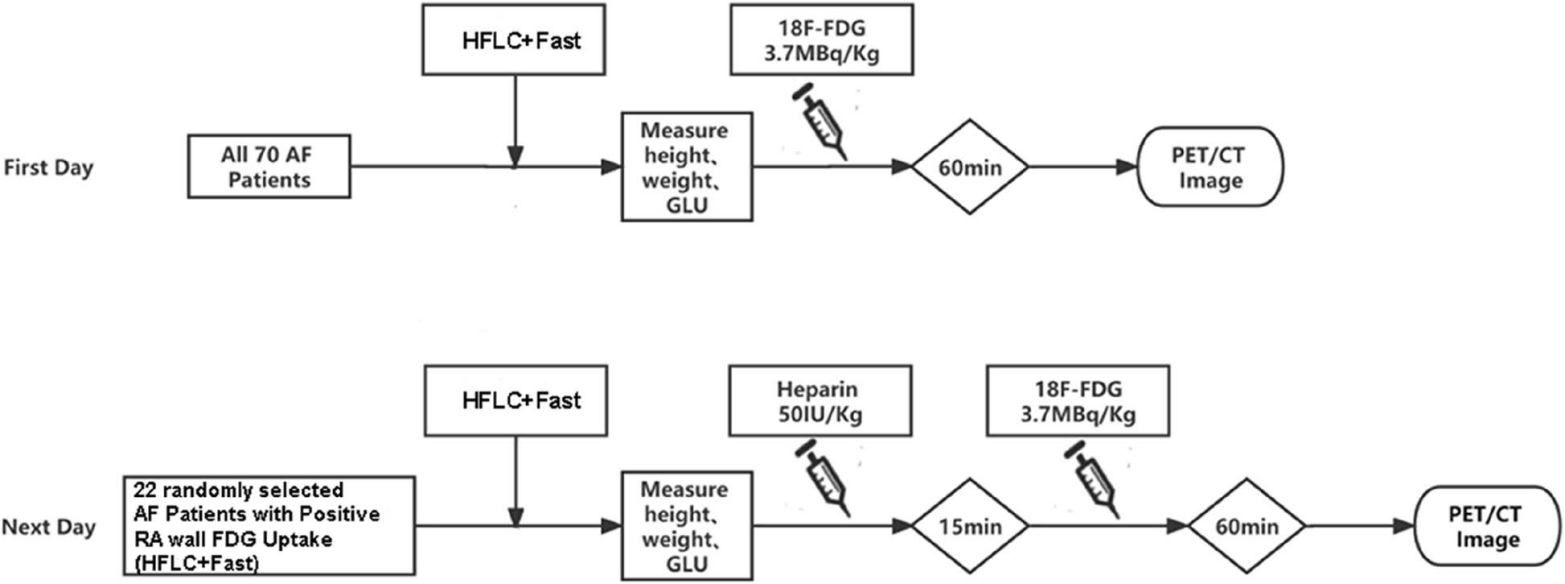
“HFLC+ Fast” method and “HFLC+ Fast+ Heparin” method Study. Road map Display

^18^F-FDG PET/CT images were obtained by Siemens Biograph mCT (64) PET/CT scanner (Siemens Medical Solutions, Knoxville, USA).^18^F-FDG was provided by Nanjing Jiangyuan Andy Cozhenge Research and Development Co., Ltd., radiochemical pure> 95%. Pre-imaging preparation: (I) high-fat and low-carbohydrate two-meal diet; (II) prolonged fast > 12 hours. A menu of permitted and banned foods and a questionnaire was given to patients to verify diet adherence. To better inhibit myocardial FDG uptake and explore the mechanisms affecting AF progression, patients with atrial positive FDG uptake were randomly selected to perform “HFLC + Fast + Heparin” method the next day, with heparin (50 IU/kg) intravenous unfractionated 15 minutes before ^18^F-FDG injection additionally (Fig. 2).

Height, weight, and fasting blood glucose were measured before examination, and if fasting blood glucose were elevated (> 7.0 mmol/l), the patients were rescheduled. ^18^F-FDG was intravenously injected at a dose of 3.7 MBq/kg. After resting for 60 minutes in a quiet and comfortable environment, the patient maintained a supine position and breathed stably. A 64-slice spiral CT scan was performed with a tube current of 35 mA, a tube voltage of 120 kV, and a collimation of 0.6 mm or 1.2 mm, depending on the selected range. Length automatically generates scan time, pitch, and bed advance speed. After the CT scan, a PET 3D acquisition was performed. The single-bed scan, which was acquired over a period of 10 minutes with the heart at the center of the field of view (FOV), allows for a more comprehensive and focused investigation of any potential metabolic activity within the heart tissue. After reconstruction, the CT, PET, and fused PET/CT images were obtained in transaxial, coronal, and sagittal planes.

### ^18^F-FDG PET/CT imaging analysis

According to the PET, CT, and fused PET/CT images, the visual and quantitative analysis of the image were performed using workstations (TrueD software) twice by observer WB and YWJ for the evaluation of intra- and inter-observer reproducibility, who were well trained and experienced. Both observers were blinded to patients’ medical records. For visual analysis, a third experienced physician was consulted when the result was different; For quantitative analysis, the result is equal to the average of the measurements taken by two physicians.

In visual analysis, a 4-point grading system was used in atrial wall ^18^F-FDG uptake: grade 0, atrial wall ^18^F-FDG uptake was lower than the adjacent blood pool (background); grade 1, atrial wall ^18^F-FDG uptake was similar to the background; grade 2, atrial wall ^18^F-FDG uptake was slightly higher than the background; grade 3, atrial wall ^18^F-FDG uptake was evidently higher than the background [10]. Grade 2–3 atrial wall ^18^F-FDG uptake was defined as positive FDG uptake.

In atrial wall FDG uptake quantitative analysis [15], the FDG SUV_max_ was selected to represent the activity of right atrium and left atrium. If the atrial wall FDG uptake was negative, three circular regions of interest (ROI) with a diameter of 5 mm were placed on the wall of the atrial through the fused PET/CT images. After placing the ROIs, we then collected the FDG SUVmax values from each of these ROIs. Instead of taking only one of the measurements or selecting an arbitrary one, we then computed an average of these three measurements. To obtain the background value of ^18^F-FDG uptake, a circular ROI with a diameter of 5 mm was placed on the left atrial (LA) and right atrial (RA) blood pool, then record the mean SUV (SUV_mean_). Thereafter, a target-to-background ratio (TBR) was calculated for right and left atria by dividing the SUVmax of the ROI by the SUVmean of blood pool, respectively.

EAT is defined as the adipose tissue between the myocardium and the pericardium with a window width of − 200 to -50HU in CT images [16]. Through the fused PET/CT images, 3 circular ROIs with a diameter of 5 mm were placed in the adipose tissue near the roof of RCA, then measuring its SUV_max_, and the average of the three measurements is taken as the final result [15]. On the workstation (TrueD software), EAT was manually delineated from the pulmonary artery bifurcation to the level of the diaphragm, delineate layer by layer at 5 mm intervals, and finally click the volume calculation button to obtain the total EAT volume (V-EAT) [16]. The final value of the above measurement index was the average value measured by two physicians.

### Echocardiography

Echocardiographic images were obtained using a Philips EPIQ 7C color Doppler ultrasound system with an X5–1 probe at a frequency of 1–5 MHz. All echocardiographic images were acquired in accordance with the guidelines of the American Society of echocardiography [17].

The patient was placed in the left lateral decubitus position, kept breathing calmly, synchronously recorded the electrocardiogram to obtain the heart rate (HR) and determined the phase, and took the average value of 3–5 consecutive cardiac cycles. The left atrial diameter (LAD) and right atrial diameter (RAD) were measured in the long-axis view of the left ventricle of the sternum; left atrial volume (LAV) was obtained from apical four-chamber and two-chamber views and left ventricular ejection fraction (LVEF) was detected by biplane Simpson method.

### Hematological test

After admission, plasma markers were measured for all patients in fasting state, including triglyceride (TG), total cholesterol (TC), high-density lipoprotein cholesterol (HDL-C), low-density lipoprotein cholesterol (LDL-C), white blood cell count (WBC), red blood cell count (RBC), hemoglobin (Hb), platelet count (PLT), platelet distribution width (PDW), neutrophil, lymphocyte, C-reactive protein (CRP).

### Statistical analysis

SPSS Statistics (Version 25; IBM) and GraphPad Prism (Version 8; GraphPad Software) were used to perform the statistical analysis. The Kolmogorov-Smirnov test was used to assess the normality of the distribution of continuous variables. Continuous variables were presented as mean ± standard deviation (SD) or median (25th–75th percentile) and compared using Student’s T-test or Mann-Whitney U test. Categorical variables were presented as percentages and compared using Chi-square test or Fisher’s exact test. Spearman correlations were used to quantify correlation between not only atrial ^18^F-FDG uptake and EAT ^18^F-FDGactivity, but also RA FDG uptake and CHA2DS2-VAS score. To explore the influencing factors of PerAF, a binary logistic regression model was used. All variables with *p*-value < 0.1 in univariate logistic regression analysis were enrolled in multivariate logistic regression analysis. Odds ratio (OR) and 95% confidence intervals (CI) were calculated.

Using a complete-randomization strategy based on a computer-generated random-number table, half patients with positive RA wall FDG uptake were randomly selected and underwent a second PET/CT examination with extra heparin to further inhibit myocardial FDG uptake. RA wall FDG uptake between “HFLC + Fast” method and “HFLC + Fast + Heparin” method was compared with stacked column chart and Wilcoxon signed ranks test. Intra-and inter-observer reproducibility of FDG uptake measurement were assessed using the intraclass correlation coefficient (ICC). *P*-value < 0.05 was considered statistically significant.

## Results

### Characteristics of the study population

In total, there are 70 AF patients enrolled, including 35 PerAF patients and 35ParAF patients with age- and sex- matched. The mean age of all patients was 65 ± 10 years old, 42 (60.0%) were male. Besides, 35 patients who were admitted during the same period with sinus rhythm, no history of AF or other cardiovascular diseases were included in the control group. The comparison between PerAF, ParAF and control were shown in Table 1.

**Table 1:** Characteristics of the study population

	Control (*n* = 35)	AF (*n* = 70)	ParAF (*n* = 35)	PerAF (*n* = 35)
**Demographic parameters**				
Age, years	64.29 ± 9.82	65.23 ± 9.92	64.43 ± 10.90	66.03 ± 8.91
Male (%)	21 (60.0)	42 (60.0)	22 (62.9)	20 (57.1)
BMI, kg/m^2^	24.92 ± 2.58	25.21 ± 3.29	24.35 ± 3.10	26.06 ± 3.30^**^**^
Smoking (%)	11 (31.4)	23 (32.9)	11 (31.4)	12 (34.3)
Drinking (%)	6 (17.1)	13 (18.6)	6 (17.1)	7 (20.0)
Course of AF,months	NA	12.0 (2.5–36.0)	12.0 (2.0–24.0)	12.0 (5.0–36.0)
Hypertension (%)	18 (51.4)	43 (62.3)	21 (60.0)	22 (64.7)
Diabetes (%)	5 (14.3)	7 (10.0)	4(11.4)	3 (8.6)
Hyperlipidemia (%)	15 (44.1)	25 (35.7)	16 (45.7)	9 (25.7)
**Hematological parameters**				
Glu, mmol/L	5.89 ± 0.85	5.85 ± 0.82	5.83 ± 0.97	5.88 ± 0.64
TG, mmol/L	1.36 (1.09–1.90)	1.40(1.00–1.84)	1.63(1.02–1.96)	1.27(0.99–1.59)
TC, mmol/L	4.53 ± 0.88	4.44 ± 0.97	4.59 ± 1.00	4.29 ± 0.92
HDL, mmol/L	1.13 ± 0.39	1.07 ± 0.24	1.11 ± 0.25	1.03 ± 0.24
LDL, mmol/L	2.67 ± 0.63	2.61 ± 0.82	2.71 ± 0.88	2.50 ± 0.77
WBC, 10^9^/L	6.20 (5.67–6.84)	5.95(4.98–6.97)	5.74(5.02–7.05)	5.96(4.67–6.88)
RBC, 10^12^/L	4.39 ± 0.49	4.50 ± 0.56	4.44 ± 0.65	4.56 ± 0.47
Hb, g/L	135.06 ± 15.12	138.51 ± 18.46	136.54 ± 20.53	140.49 ± 16.19
PLT, 10^9^/L	198.43 ± 54.43	194.73 ± 55.13	200.17 ± 60.27	189.29 ± 49.74
PDW	12.80 (11.90–14.30)	13.20(11.88–14.68)	13.30(11.90–15.20)	13.20(11.80–14.20)
Neutrophil, 10^9^/L	3.64 (2.96–4.35)	3.52(2.89–4.49)	3.86(2.87–4.64)	3.31(2.89–4.38)
Lymphocyte, 10^9^/L	1.74 (1.27–1.97)	1.63(1.25–2.07)	1.64(1.13–2.04)	1.62(1.29–2.17)
CRP, mg/L	3.80 (3.00–4.68)	3.50(3.10–4.43)	3.50(3.10–4.20)	3.80(3.10–4.80)
**ECG parameters**				
HR	77.66 ± 11.12	82.71 ± 18.52	74.89 ± 14.86	90.54 ± 18.69^**‡^**^
AXIS	40.46 ± 20.66	37.56 ± 37.39	32.17 ± 32.92	42.94 ± 41.15
QRS wave duration	103.00 (93.00–112.00)	100.00(94.75–106.25)	98.00(94.00–96.00)	100.00(96.00–108.00)
**Echo parameters**				
LAD,mm	34.94 ± 3.95	42.40 ± 6.40^*^	38.40 ± 5.81 ^**†**^	46.40 ± 4.07^**‡^**^
LVEF	63.63 ± 2.30	61.00 ± 4.90 ^*^	62.97 ± 4.08	59.03 ± 4.90 ^**†^**^
RAD,mm	29.80 ± 2.73	39.91 ± 6.69^*^	35.31 ± 4.72 ^**†**^	44.51 ± 4.99^**‡^**^

*AF* atrial fibrillation, *BMI* body mass index, *SAS* sleep apnea syndrome, *Glu* blood glucose, *TG* triglyceride, *TC* total cholesterol, *HDL* high-density lipoprotein, *LDL* low-density lipoprotein, *WBC* white blood cell count, *RBC* red blood cell count, *CRP* c-reactive protein, *HR* heart rate, *LAD* left atrial diameter, *LVEF* left ventricular ejection fraction, *RAD* right atrial diameter

^*^P-value < 0.05 AF compared with control; ^**†**^*P*-value < 0.05 ParAF compared with control; ^**†**^*P*-value < 0.05 PerAF compared with control; ^**^**^*P*-value < 0.05 PerAF compared with ParAF

Compared with ParAF group, the BMI was significantly higher in PerAF group (*P* = 0.029). In ECG parameters, the HR of PerAF group had higher HR than ParAF group (*P* < 0.001). In the echocardiographic parameters, the LAD and RAD in the PerAF group were higher than ParAF group, the LVEF was lower than that of the ParAF group significantly (all *P* < 0.05). There were 37 patients had BNP results. PerAF group had significantly higher BNP than ParAF group [135 (87, 227) vs, 49 (10, 141) *P* = 0.003].

### Comparison of visual analysis and quantitative analysis of RA wall FDG uptake in PerAF and ParAF

PerAF patients had significantly higher probability of RA wall FDG positive uptake than ParAF group by visual analysis and quantitative analysis (both *P* < 0.05). The EAT SUV_max_ and V-EAT in PerAF group were significantly higher than those in ParAF group (*P* < 0.05) (Table 2). Besides, AF patients had significantly higher probability of RA wall FDG positive uptake, SUV_max_ and TBR than control group (all *P* < 0.05).

**Table 2:** Comparison of ^18^F-FDG PET/CT parameters in ParAF, PerAF and control group

	Control (*n* = 35)	AF (*n* = 70)	ParAF (*n* = 35)	PerAF (*n* = 35)
**Visual analysis**				
LA wall positive FDG uptake (%)	1(2.9)	20(28.6)^*^	5(14.3) ^**†**^	15(42.9)^**‡^**^
RA wall positiveFDG uptake (%)	1(2.9)	42(60.0)^*^	10(28.6) ^**†**^	32(91.4)^**‡^**^
**Quantitative analysis**				
SUV_max_				
LA SUV_max_	2.10(1.90–2.60)	2.85(2.48–3.53) ^*^	2.60(2.30–3.00) ^**†**^	3.40(2.70–4.00)^**‡^**^
RA wall SUVmax	2.15(1.70–2.40)	3.10(2.60–4.20) ^*^	2.60(2.40–3.10) ^**†**^	4.10(3.20–4.90)^**‡^**^
Spleen SUVmax	2.71(2.44, 3.21)	2.89 (2.60, 3.02)	2.87(2.57, 3.20)	2.94(2.69, 3.21)
Marrow SUVmax	3.12(2.66, 3.47)	3.31 (2.86, 3.77)	3.23(2.68, 3.92)	3.34(3.08, 3.65)
TBR				
LATBR	1.30(0.88–1.57)	1.35(1.24–1.85) ^*^	1.30(1.21–1.44)	1.45(1.26–2.00)^**‡^**^
RATBR	1.38(0.79–1.54)	1.49(1.30–2.05) ^*^	1.35(1.25–1.50)	1.89(1.45–2.67)^**‡^**^
EAT				
EAT SUV_max_	1.07 ± 0.29	1.51 ± 0.37^*^	1.42 ± 0.34 ^**†**^	1.59 ± 0.38^**‡**^
V-EAT,cm^3^	93.18 ± 35.25	137.94 ± 49.78^*^	126.01 ± 56.95 ^**†**^	149.88 ± 38.63^**‡^**^

*AF* atrial fibrillation, *LA* left atrial, *RA* right atrial, *LV* left ventricle, RV right ventricle, SUV_max_ maximum standardizeduptake value, *LAA* left atrial appendage, *RAA* right atrial appendage, *TBR* target-to-background ratio, *EAT* epicardial adipose tissue, *V-EAT* total epicardial adipose tissue volume

^*^P-value < 0.05 AF compared with control; ^**†**^*P*-value < 0.05 ParAF compared with control;^**‡**^*P*-value < 0.05 PerAF compared with control;^**^**^*P*-value < 0.05 PerAF compared with ParAF

### Univariate and multivariate analysis of factors affecting the PerAF

In 70 AF patients, results are shown for univariate and multiple regression analysis with PerAF (dependent variable). Upon conducting a univariate analysis for PerAF, substantial correlations were found with variables including BMI, LAD, LVEF, RAD, LA wall SUVmax, RA wall SUVmax, EAT SUVmax, and V-EAT (*P* all < 0.1).

The variables with statistical significance in the univariate analysis were included in the multivariate logistic regression model, and RA wall SUVmax was the only independent variable for PerAF (OR = 1.804, 95%CI 1.023–3.182, *P* = 0.041) (Table 3).

**Table 3:** Univariate and multivariate analysis of factors affecting PerAF

Variable	Univariate analysis		Multivariate analysis	
	OR (95% CI)	*P*-value	OR (95% CI)	*P*-value
BMI	1.19(1.01–1.40)	0.035	0.76(0.41–1.38)	0.36
RAD	1.42(1.22–1.65)	<0.001	1.19(0.95–1.48)	0.13
LAD	1.42(1.20–1.68)	<0.001	1.70(0.97–2.99)	0.06
LVEF	0.82(0.72–0.93)	0.002	0.87(0.63–1.19)	0.38
RA wall SUVmax	1.34(1.15–1.55)	<0.001	1.80(1.02–3.18)	0.04
LA wall SUV_max_	1.11(1.03–1.12)	0.003	0.76(0.50–1.16)	0.21
EAT SUV_max_	1.21(1.03–1.43)	0.02	1.13(0.69–1.842)	0.62
V-EAT (cm^3^)	1.01(1.00–1.02)	0.05	0.99(0.96–1.02)	0.60

*BMI* Body Mass Index, *RAD* right atrial diameter, *LAD* left atrial diameter, *LVEF* left ventricular ejection fraction, RA wall SUVmax maximum standardized uptake value of right atrial, LA *SUVmax* maximum standardized uptake value of left atrial, *EAT SUVmax* maximum standardized uptake value of epicardial adipose tissue, *V-EAT* volume of epicardial adipose tissue

### Influencing factors of RA wall FDG positive uptake in AF patients

In 70 AF patients, results are shown for univariate and multiple regression analysis with RA wall FDG positive uptake (dependent variable). PerAF (OR: 7.26, 95% CI: 1.04–50.45, P = 0.04) and EAT SUVmax (OR: 1.39, 95% CI: 1.10–1.75, *P* = 0.005) were independently related to RA wall FDG positive uptake after adjusting for confounding factors (Table 4).

**Table 4:** Influencing factors of RA wall FDG positive uptake in AF patients

	Univariate analysis		Multivariate analysis	
	OR (95% CI)	*P* value	OR (95% CI)	*P*-value
PerAF	10.33 (3.00, 35.63)	<0.001*	7.26 (1.04, 50.45)	0.04*
EAT SUV_max_	1.39 (1.14, 1.69)	0.001*	1.39 (1.10, 1.75)	0.005*

*EAT SUVmax* maximum standardized uptake value of right coronary artery epicardial adipose tissue, *RA* right atrial, OR (95%CI): Odds Ratio (95% Confidence Interval); *AF* atrial fibrillation; *FDG* Fluorodeoxyglucose; *PerAF* persistent AF; *P value ＜0.05

### Comparison between HFLC + fast and HFLC + fast + heparin method in positive RA wall FDG uptake AF patients

Among the 22 AF cases with positive RA wall FDG uptake (HFLC + Fast method) received “HFLC+ Fast+ Heparin” method the next day. In comparison to the HFLC + Fast methodology, the uptake of FDG in the RA wall among patients remained unaltered under the application of the HFLC + Fast + Heparin method [RA wall SUVmax: 4.15 (3.18–4.90) VS. 3.90 (3.10–5.70), *P* = 0.896; RA wall positive uptake: 100% (22/22) VS. 86.4% (19/22), *P* = 0.233] (Table 5).

**Table 5:** Comparation between HFLC + Fast and HFLC + Fast + Heparin method in positive atrial FDG uptake AF patients by visual and quantitative analysis

visual analysis		HFLC+ Fast	HFLC+ Fast+ Heparin	*P*-value
visual analysis	RA wall positive FDG uptake	100%(22/22)	86.4% (19/22)	0.233
	LA wall positive FDG uptake	54.5%(12/22)	45.5%(10/22)	0.763
quantitative analysis	LA wall SUV_max_	3.55 (2.58–4.48)	3.00 (2.48–4.30)	0.086
	RA wall SUVmax	4.15 (3.18–4.90)	3.90 (3.10–5.70)	0.896

*AF* atrial fibrillation, *LA* left atrial, RA:right atrial, *FDG* fluorodeoxyglucose, *SUVmax* maximum standardized uptake value

### The relationship between CHA2DS2-VAS score and RA wall FDG uptake

Spearman correlation analysis showed the weak association between RA wall FDG uptake and CHA2DS2-VAS score (Spearman correlation coefficient: *r* = 0.30, *P* = 0.01). High CHA2DS2-VASc score group had significantly higher RA wall SUVmax than low CHA2DS2-VASc score group [3.35 (2.70, 4.50) vs, 2.8 (2.4, 3.1) *P* = 0.01].

### Reproducibility

Table 6 presented the reproducibility of PET/CT parameters (LA SUV_max_, RA wall SUVmax, EAT SUV_max_, V-EAT). Both intra- and inter-observer comparisons showed excellent reproducibility in all the measurements (all ICC > 0.8).

**Table 6:** Intra- and inter-observer reproducibility

	intra-observer		inter-observer	
	ICC (95% CI)	*P* value	ICC (95% CI)	*P* value
LA wall SUV_max_	0.990 (0.984–0.994)	<0.001	0.986 (0.977–0.991)	<0.001
RA wall SUV_max_	0.991 (0.986–0.996)	<0.001	0.992 (0.987–0.998)	<0.001
EAT SUV_max_	0.994 (0.990–0.998)	<0.001	0.990 (0.985–0.993)	<0.001
V-EAT	0.989 (0.983–0.994)	<0.001	0.986 (0.982–0.990)	<0.001

*ICC* intraclass correlation coefficient, *LA* left atrial, *SUV*_*max*_ maximum standardizeduptake value, *LAA* left atrial appendage, *RA* right atrial, *RAA* right atrial appendage, *EAT* epicardial adipose tissue, *SUV*_*max*_ maximum standardizeduptake value, *V-EAT* total epicardial adipose tissue volume

## Discussion

The main findings were as follows: 1. 91.4% PerAF patients exhibit RA wall FDG positive uptake. Quantitative parameters of RA wall FDG uptake (SUV_max_) was significantly higher than ParAF group. 2. Multivariable analysis showed that RA wall SUVmax was the independent risk factor for PerAF. 3. PerAF and EAT SUV_max_ was independently related to RA wall FDG positive uptake. 4. In comparison to the HFLC + Fast methodology, the RA wall FDG uptake remained unaltered among patients under the application of the HFLC + Fast + Heparin method, which further verified that RA wall FDG uptake may prefer pathological inflammation. 5. High CHA2DS2-VASc score group had higher RA wall ^18^F-FDG uptake, which indicated a higher risk of stroke.

Approximately 5.5% of ParAF progresses to PerAF each year, which had a higher risk of stoke [18]. It begins with a rapidly triggered focal drive (paroxysmal state) and maintains AF (persistent state) through the development of a functional atrial substrate, followed by atrial remodeling, which is often irreversible and accompanies a poor prognosis [19]. Therefore, early intervention before irreversible remodeling is important. Several converging lines of evidence suggest that inflammation is critical in the development of AF [20]. Inflammation leads to atrial fibrosis, gap junction regulation, and abnormal intracellular calcium handling, increasing atrial ectopic activity and slowing atrial conduction, ultimately leading to structural and electrical remodeling of the atrium [21]. Pathology showed lymphocyte cell infiltration and adjacent myocyte necrosis around the atrium in patients with AF [22]. Inflammatory mediators including TNF-α, IL-6, IL-1β, IL-8 and MPO by hematological indicators play an important role in the process of AF [23]. Chao TF et al. showed that CRP levels were significantly higher in persistent or permanent AF patients than paroxysmal AF patients, indicating that higher levels of inflammatory factors may increase the load of PerAF [24]. Another retrospective analysis results also showed that PerAF patients had higher CRP levels than ParAF, which speculated that the role of inflammation in AF maintenance may be more sense than that AF initiating [18]. Plasma inflammatory markers lack specificity and cannot fully reflect the local atrial inflammation. The inflammatory load of local organs may not be related to plasma concentrations of circulating cytokines [25]. In the present study there was no significant difference between PerAF and ParAF in plasma inflammatory markers (WBC, neutrophil, lymphocyte, and CRP). As the “incubator” for monocytes, the difference in FDG metabolic activity of hematopoietic tissue (spleen and bone marrow) also did not reach significance between PerAF and ParAF in our study. The current gold standard for evaluating inflammation is invasively pathological biopsy, which is inappropriate for dynamic evaluation of local cardiac inflammatory activity in clinical practice. It is necessary to use non-invasive imaging to evaluate local atrial inflammatory activity in AF patients.

^18^F-FDG PET/CT has been successfully employed to visualize inflammatory processes even in low-grade inflammatory diseases as in the atria of AF patients [26]. The initial changes of inflammation are mainly the rapid congestion of tissues, the increase of vascular permeability and the release of many inflammatory factors, which lead to increased blood perfusion, and promote the large and rapid uptake of ^18^F-FDG at the lesion site. Several PET/CT-based retrospective studies found that increased atrial ^18^F-FDG uptake is associated with AF, especially in RA [27, 28]. Besides, the autopsy of patients with AF found many macrophages and lymphocyte infiltration in the area of atrial ^18^F-FDG uptake [10]. This is the first study to prospectively explore the influencing factors from ParAF to PerAF based on ^18^F-FDG PET/CT. In visual and quantitative analysis, the study found that the RA FDG uptake was the independent risk factors of PerAF after adjustment for confounders. The results highlight the position of inflammation in the pathophysiology of PerAF, suggest that inflammation is mainly accumulated in the RA. The reason that right-atrial wall FDG uptake was statistically significant rather than left-atrial FDG uptake may be as follows. Firstly, AF patients display differential degrees of fibrosis between the left and the right atria, with the left atrium experiencing a more profound degree of fibrosis. This severe fibrosis is concomitant with apoptosis and eventual mortality of atrial cells, which may subsequently reduce glucose uptake in the left atrium. Besides, it could be a process of diffuse atrial remodeling related to the AF condition, which can also affect the right atrium, as structural and electrical remodeling is not limited to the left atrium. Another possibility could be related to an increased workload on the right side of the heart due to underlying conditions like pulmonary hypertension, myocardial ischemia or heart failure [29].

Our study evaluated potential factors influencing RA positive FDG uptake among AF patients. In concurrence with the findings of Xie et al., our findings not only reaffirmed the role of PerAF, but also identified EAT SUVmax as an independent determinant, even after adjusting for other confounding variables [11]. EAT is an active endocrine organ, which can produce a large number of inflammatory mediators to act on the atrium, leading to its electrical and structural remodeling, and increased ^18^F-FDG uptake in EAT is representative of or associated with inflammatory activity [30].

The atrial FDG uptake observed in patients subjected to the HFLC + Fast + Heparin method the next day did not exhibit significant alterations when compared to the HFLC + Fast method. This observation further implies that atrial FDG uptake in AF patients evaluated with 18F-FDG PET/CT, may have a predilection towards pathology as opposed to energy substrate metabolism. It was reported that ^18^F-FDG PET/CT performed under routine conditions did not detect a significant difference in artium inflammatory activity, physiological FDG uptake or myocardial energy substrate metabolism between patients with and without AF [17]. Physiological glucose uptake can be inhibited by HFLC+Fast with the decrease of blood glucose level and/or insulin level [31]. Besides, plasma FFA levels increase dramatically after heparin injection, reducing glucose consumption in normal myocardium, thus highlighting FDG uptake in inflammatory lesions [32]. To further inhibit the physiological uptake of the myocardium and explore the mechanism of atrial FDG uptake, we performed HFLC + Fast + Heparin in 22 randomly selected patients with positive atrial FDG uptake (HFLC+Fast). And the abnormal uptake, which largely reflects the activity of inflammation, further supports that inflammation is an important factor in AF progression, which is critical for anti-inflammatory treatment of AF. Therefore, the implementation of 18F-FDG PET/CT could enable the detection of localized atrial inflammatory responses. This tool could also serve as a guide for prescribing anti-inflammatory treatments, and subsequently, performing dynamic detection following post-treatment monitoring for patients with AF.

Besides, RA ^18^F-FDG uptake was weak associated with CHA2DS2-VASc score and higher in high CHA2DS2-VASc score group. CHA2DS2-VASc score is well-recognized score for stratification of stroke risk in AF patients. It was reported that the rate of stroke increased with higher CHA2DS2-VASc score, which required anticoagulant therapy [33]. Aretrospective study concluded that stroke was strongly associated with RA positive uptake in multivariate analysis [34]. Our previous study concluded that RA FDG uptake improves prediction of stroke above the CHA2DS2-VASc score in patients with AF [35].

This study had certain limitations. Firstly, as a single-center study with small sample size, it requires a large sample study for validation. Secondly, the present study did not perform invasive pathological biopsy of ^18^F-FDG-uptake tissue in the atria and lacked the gold standard for diagnosing inflammation. Finally, the study lacked follow-up data that might indicate the prognostic significance of RA FDG uptake.

## Conclusions

RA wall FDG positive uptake was present mainly in PerAF. RA wall ^18^F-FDG uptake was an independent influencing factor of PerAF. RA FDG uptake based on ^18^F-FDG PET/CT may prefer pathological inflammation.

## Data Availability

The datasets generated and/or analysed during the current study are not publicly available due to departmental management, but are available from the corresponding author on reasonable request.
